# Economic evaluation of first-line sugemalimab plus chemotherapy for metastatic non-small cell lung cancer in China

**DOI:** 10.3389/fonc.2022.1081750

**Published:** 2022-12-13

**Authors:** Hao Wang, Li Liao, Yuan Xu, Yunchun Long, Ye Wang, Yujie Zhou

**Affiliations:** ^1^ Department of Pharmacy, Nanjing Drum Tower Hospital, The Affiliated Hospital of Nanjing University Medical School, Nanjing, China; ^2^ School of Basic Medical Sciences and Clinical Pharmacy, China Pharmaceutical University Nanjing Drum Tower Hospital, Nanjing, China; ^3^ Department of Respiratory and Critical Care Medicine, Nanjing Drum Tower Hospital, The Affiliated Hospital of Nanjing University Medical School, Nanjing, China

**Keywords:** sugemalimab, chemotherapy, metastatic non-small cell lung cancer, cost-utility analysis, economic evaluation, partitioned survival model

## Abstract

**Objective:**

To evaluate the economics of sugemalimab plus chemotherapy in the first-line treatment of metastatic non-small cell lung cancer, and to provide a reference for the formulation of relevant medical insurance policies and rational drug use.

**Methods:**

From the perspective of the Chinese health system, a three-state partitioned survival model was constructed based on data from a phase III randomized clinical trial (GEMSTONE 302) to evaluate the cost-utility of sugemalimab plus chemotherapy compared with chemotherapy in first-line treatment of metastatic non-small cell lung cancer. Model results were expressed as total cost, life years, quality-adjusted life years, and incremental cost-effectiveness ratio. The robustness of the underlying analysis results was verified using one-way sensitivity analysis and probabilistic sensitivity analysis.

**Results:**

The results of the base-case analysis showed that sugemalimab plus chemotherapy yielded 1.63 QALYs at a total cost of 130,667.70 USD, chemotherapy yielded 1.04 QALYs at a total cost of 64,001.02 USD, and the ICER was 113,155.52 USD/QALY, which was well above the current willingness-to-pay threshold in China (3 times 2021 per capita GDP) (36,203.88 USD).

**Conclusion:**

This study suggests that sugemalimab in combination with a chemotherapy regimen is more effective but not economical for patients with metastatic non-small cell lung cancer receiving first-line therapy in China and that a reasonable reduction in drug prices could improve the probability of it being economical.

## Introduction

Lung cancer is the most common malignant tumor of the respiratory system. About 80-85% of subtypes are non-small cell lung cancer (NSCLC). According to histology, NSCLC can be classified as adenocarcinoma (40%), squamous carcinoma (20%), large cell carcinoma, and mixed tissue tumors (1, 2).

In 2020, there were 2.2 million new lung cancer patients worldwide, accounting for 11.4% of new cancers, and 1.79 million deaths, accounting for 18% of cancer-related deaths, making it the second most common cancer and the first cause of cancer-related deaths in the world, while China ranks first in the world in both incidence and mortality of lung cancer, which brings a great economic burden to the society (3). A projection study based on the GLOBOCAN 2020 and the 2019 China Cancer Registry Reports indicates that lung cancer incidence and mortality rates in China have increased due to increasing population size and aging. By 2022, the number of new lung cancer cases and deaths in China will increase to 870,982 and 766,898, respectively. The burden associated with lung cancer also has increased (4). A study showed that the economic burden of lung cancer in China in 2017 was approximately $25.069 billion (0.121% of the gross national product in that year), with an estimated direct expenditure of $11.098 billion, or 1.43% of total health expenditure in China. Based on current lung cancer incidence projections, the economic burden in China will increase to $40.4 billion and $53.4 billion by 2025 and 2030, respectively, accounting for 0.121% and 0.131% of gross domestic product (GDP), respectively (5, 6).

NSCLC is usually diagnosed when metastatic disease is already present and the best time for surgery is missed. Previously, the standard treatment regimen for patients with metastatic NSCLC (mNSCLC) was chemotherapy with cytotoxic agents, with a poor prognosis and a five-year survival rate of less than 5%. In recent years, immune checkpoint inhibitors (ICIs) have emerged as a new treatment strategy for patients with mNSCLC without driver mutations (EGFR/ALK/ROS 1, etc.). ICIs targeting the programmed cell death protein 1 (PD-1)/programmed cell death ligand 1 (PD-L1) pathway are among the most important immunotherapeutic agents in the treatment of mNSCLC (7).

Blocking the binding of PD-1 expressed in tumor-infiltrating T lymphocytes to PD-L1 expressed in tumor cells can partially reverse T cell dysfunction and inhibit tumor growth (8–10). Both PD-1/PD-L1 inhibitors can block PD-1/PD-L1 interactions and exert anti-tumor effects. Compared with PD-1 inhibitors, PD-L1 inhibitors can bind PD-L1 on the surface of both tumor cells and antigen-presenting cells, which is conducive to the full activation of T cells and restoration of T cell-mediated antitumor immunity, with stronger overall immune efficacy and without affecting the physiological function of PD-L2. PD-1/PD-L1 inhibitors alone quickly develop drug resistance; combining with chemotherapy can enhance anti-tumor immunity by inducing immunogenic cell death, improving efficacy, and delaying the onset of drug resistance (2, 11, 12).

Sugemalimab (Cejemly^®^ in China), the first PD-L1 inhibitor for mNSCLC developed independently in China, is a high-affinity, fully humanized, full-length anti-PD-L1 IgG4 monoclonal antibody that selectively binds PD-L1 and blocks its interaction with PD-1 and the leukocyte differentiation antigen CD80 to exert anti-tumor effects. On December 12, 2021, based on the GEMSTONE 302 trial (NCT03789604), sugemalimab was approved in China for the first-line treatment of EGFR/ALK-negative mNSCLC in combination with pemetrexed and carboplatin for non-squamous NSCLC, and in combination with paclitaxel and carboplatin for squamous NSCLC (13, 14). The GEMSTONE 302 trial was a double-blind, randomized, phase III clinical trial that evaluated the efficacy and safety of sugemalimab in combination with chemotherapy in patients with squamous or non-squamous mNSCLC. Compared with chemotherapy alone, sugemalimab plus chemotherapy significantly prolonged median progression-free survival (9.0 months vs. 4.9 months), with a trend toward significantly benefit in overall survival (22.8 months vs. 17.7 months), a 33% lower risk of death, and a lower rate of grade 3-5 treatment-related adverse events (22.8% vs. 19.5%). The Chinese Society of Clinical Oncology guidelines for the treatment of NSCLC have included sugemalimab as a first-line treatment for advanced NSCLC in 2022.

Sugemalimab plus chemotherapy provides more survival benefits to patients but is expensive post-marketing [1844.26 USD/(600mg/20ml)], with a price of 3688.52 USD for one cycle of the immune drug alone. Therefore, it is equally important to clarify the economics of sugemalimab plus chemotherapy. The purpose of this study was to evaluate the cost-utility of sugemalimab plus chemotherapy compared with chemotherapy in the first-line treatment of mNSCLC and to provide some references for healthcare decisions and rational clinical use of the drug.

## Materials and methods

To develop a partitioned survival model to assess the cost-utility of sugemalimab plus chemotherapy in the first-line treatment of mNSCLC using TreeAge Pro 2022. From the Chinese health system perspective, only direct medical costs were included, all expressed in United States dollars (USD) (1 USD=CNY ¥6.71). Patient survival data, dosing regimens, subsequent therapy after disease progression, and incidence of serious adverse events (SAEs) were obtained from the GEMSTONE 302 trial. Model outcomes were expressed as total cost, life years (LYs), quality-adjusted life years (QALYs), and incremental cost-effectiveness ratio (ICER). According to *the Chinese pharmacoeconomic evaluation guidelines* (2020 version), an annual discount rate of 5% was used for both cost and utility. The willingness-to-pay (WTP) threshold was defined as three times the GDP per capita in China in 2021 (36,203.88 USD/QALY), as recommended by the World Health Organization (15, 16).

### Model structure

**Figure 1:**
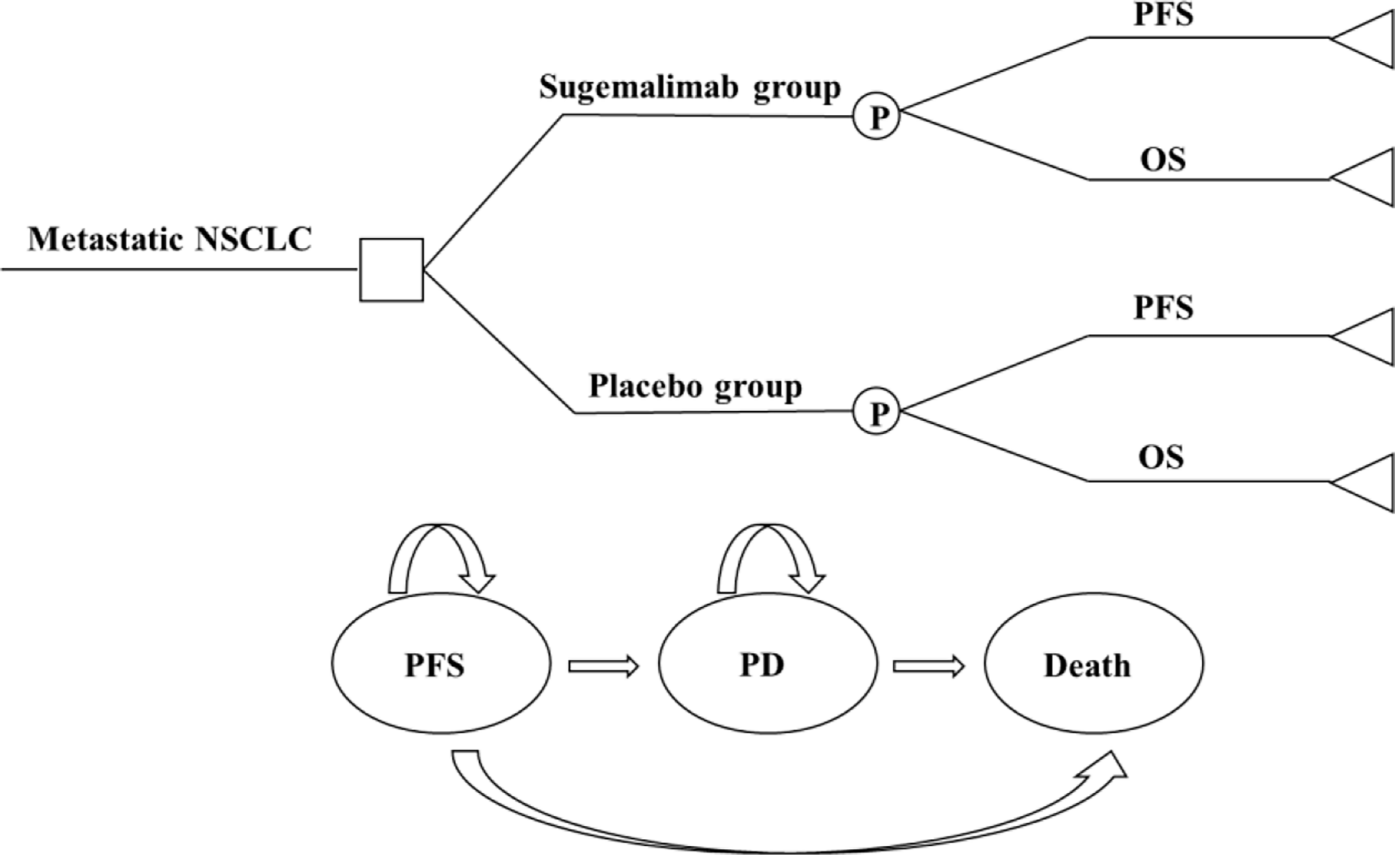
A three-state partitioned survival analysis (PartSA) model simulating metastatic non-small cell lung cancer. Sugemalimab group, sugemalimab + platinum-based chemotherapy; Placebo group, placebo + platinum-based chemotherapy: NSCLC, non-small cell lung cancer; PFS, progression-free-survival; PD, progressive disease; OS, overall survival.

The model included three mutually exclusive health states: progression-free survival (PFS), progressive disease (PD), and death. All patients had an initial state of PFS and an end state of death (**Figure 1**). The model cycle was consistent with the drug administration cycle (21 days). The model run cycle was performed for 10 years (99% of patients die within 10 years). The disutility generated by SAEs was deducted in the first cycle of the model run.

### Clinical data

The target population in this study was consistent with the GEMSTONE 302 trial. Eligible patients were aged 18-75 years, had histologically or cytologically confirmed stage IV non-squamous or squamous NSCLC without driver genes mutation such as EGFR/ALK/ROS 1, and had received no previous systemic treatment for metastatic disease. Patients with at least one measurable lesion according to Response Evaluation Criteria in Solid Tumors (RECIST), version 1.1, an Eastern Cooperative Oncology Group (ECOG) performance status (PS) of 0 or 1.

The dosing regimens for the two interventions in this study were sugemalimab (1200 mg by intravenous infusion [IV] every 3 weeks) combined with platinum-based chemotherapy (squamous: carboplatin AUC=5 mg/mL/min, IV and paclitaxel 175 mg/m^2^, IV; nonsquamous: carboplatin AUC=5 mg/mL/min, IV and pemetrexed 500 mg/m^2^, IV) (sugemalimab group) or placebo combined with platinum-based chemotherapy (as above) (placebo group); after 4 cycles of treatment, patients with squamous NSCLC received sugemalimab or placebo maintenance therapy and patients with non-squamous NSCLC received sugemalimab combined with pemetrexed (500 mg/m^2^ IV) or placebo combined with pemetrexed (500 mg/m^2^ IV) maintenance therapy until disease progression or intolerable adverse reactions or to 35 cycles.

The limited follow-up time in clinical trials makes it difficult to track the effect of drug administration on the target population over time. Therefore, this study used the Engauge Digitizer software (http://digitizer.sourceforge.net) to extract individual patient data from Kaplan-Meier (K-M) curves of PFS and OS in the GEMSTONE 302 trial. Curve fitting and extrapolation were performed in R software (version 4.2.1; https://www.r-project.org) using standard parametric models (exponential, Weibull, Gompertz, log-logistic, and log-normal models). The best-fit distribution was selected by visual inspection combined with goodness-of-fit tests [Akaike information criterion (AIC) and Bayesian information criterion (BIC)].

### Cost

Only direct medical costs were included in this study, including costs of medications, routine follow-up, hospitalization, management of SAEs, and subsequent therapy of disease progression.

Drug costs were calculated based on the dosing regimen in the GEMSTONE 302 trial, and all drugs were priced using an average of the winning bid from the YAOZH (https://www.yaozh.com/).

Patients undergo regular imaging (CT/PET-CT) and laboratory examinations during treatment. The fees for the relevant examinations (routine blood, urine, stool, biochemistry, thyroid function tests, five coagulation tests, electrocardiogram, five serum lung cancer tests, etc.) and all medical expenses incurred during hospitalization (bed fees, consultation fees, nursing fees, intravenous infusion fees, and chemical dispensing fees) were based on the medical service prices in Jiangsu Province.

Only grade 3-5 adverse events with an incidence of ≥10%, i.e., decreased neutrophil count, nausea, vomiting, alopecia, rash, fatigue, and diarrhea, were included in this study, as grade 1-2 adverse reactions usually do not require treatment.

In the GEMSTONE 302 trial, after disease progression, patients were treated partly with sugemalimab monotherapy, partly with new antitumor therapy (treatment regimen not provided, assuming all were treated with docetaxel), and the remaining patients received only the best supportive care (17, 18).

In this study, a creatinine clearance (GFR) of 70 ml/min and a body surface area of 1.72 m^2^ was assumed to calculate the drug dose, and the dose of carboplatin was calculated using the Calvert formula = AUC*(GFR+25) (19, 20).

### Health utility

Patient utility values were not reported in the GEMSTONE 302 trial, and the health utility values in this study were taken from a Chinese population in a multi-regional study of health utility values. The health utility values were 0.804 for PFS and 0.321 for PD (21).

### Sensitivity analysis

To assess the robustness of the model results, one-way sensitivity analysis and probabilistic sensitivity analysis (PSA) were conducted in this study.

In the one-way sensitivity analysis, the lower limit of sugemalimab was set to 50% of the current price and the upper limit to the current price; the range of utility values for different disease states and adverse effects were taken as the maximum and minimum values for different country populations; the range of discount rate was set to 0-8% and the range of other parameters not specifically stated fluctuated above and below their base values ±20%. To test the effect of each parameter on the model results, and the results were represented as tornado plots.

The PSA was run 1,000 times using the second order-Monte Carlo simulation. The gamma distribution was used for all cost parameters, and the beta distribution was used for health utility values and the incidence of adverse events. The incremental cost-effectiveness scatter plots and cost-effectiveness acceptability curve (CEAC) of the simulation results were used to determine the probability that the 2 interventions were economical at different WTP thresholds.

### Scenario analysis

Three different scenarios were considered in this study: (1) to investigate the effect of the price of sugemalimab on the economics of sugemalimab combination chemotherapy regimen, the price of sugemalimab was reduced by 70% and 90%; (2) the simulation time frame of the model was set to 5 and 20 years to assess the consumption of medical costs and survival benefit of mNSCLC patients throughout the disease course; (3) the OS curves were extrapolated using a mixture cure model, a nonmixture cure model, and Royston/Parmar spline model to fit extrapolation of OS curves in the sugemalimab and placebo groups to assess the effect of different model fits and extrapolation of survival curves on the results.

The details of the base values, ranges, and distribution of the model parameters were shown in **Table 1**.

**Table 1:** Key model inputs.

Parameters	Base-case Values	Ranges	Distribution	Reference
Cost (USD)				
Sugemalimab per 600mg	1844.26	922.13-1844.26	gamma	yaozh
Carboplatin per 100mg	13.39	10.71-16.06	gamma	yaozh
Pemetrexed per 100mg	330.23	264.18-396.27	gamma	yaozh
Paclitaxel per 30mg	51.39	41.12-61.67	gamma	yaozh
Docetaxel per 20mg	96.65	77.32-115.97	gamma	yaozh
Hospital management per cycle	72.43	57.94-86.91	gamma	Local charge
Follow-up per cycle	199.55	159.64-239.46	gamma	Local charge
SAEs of sugemalimab group	23.57	18.86-28.28	gamma	Local charge
SAEs of placebo group	23.81	19.05-28.57	gamma	Local charge
Best supportive care per cycle	320.84	256.67-385.01	gamma	(18)
Probability of SAEs (grade ≥ 3)				
Neutropenia of sugemalimab group	0.3250	0.2600-0.3900	beta	(14)
Rnausea and vomiting of sugemalimab group	0.0160	0.0128-0.01920	beta	(14)
Alopecia of sugemalimab group	0.0030	0.0024-0.0036	beta	(14)
Rash of sugemalimab group	0.0060	0.0048-0.0072	beta	(14)
Fatigue of sugemalimab group	0.0090	0.0072-0.1080	beta	(14)
Diarrhoea of sugemalimab group	0.0090	0.0072-0.1080	beta	(14)
Neutropenia of placebo group	0.3330	0.2664-0.3996	beta	(14)
Nausea and vomiting of placebo group	0.0250	0.0200-0.0300	beta	(14)
Fatigue of placebo group	0.0060	0.0048-0.0072	beta	(14)
Utility values				
PFS	0.804	0.536-0.840	beta	(21)
PD	0.321	0.031-0.473	beta	(21)
Disutility values				
Neutropenia	-0.20	-0.50 to -0.15	beta	(21)
Nausea and vomiting	-0.12	-0.29 to -0.06	beta	(21)
Alopecia	-0.06	-0.26 to -0.06	beta	(21)
Rash	-0.10	-0.19 to -0.10	beta	(21)
Fatigue	-0.07	-0.49 to -0.07	beta	(21)
Diarrhoea	-0.07	-0.35 to -0.06	beta	(21)
Subsequent treatment proportion of sugemalimab group				
New Anti-Cancer Therapy	53.10%	–	–	(14)
Open-label Sugemalimab in crossover	5.6%	–	–	(14)
Others	41.3%	–	–	(14)
Subsequent treatment proportion of placebo group				
New Anti-Cancer Therapy	25.10%	–	–	(14)
Open-label Sugemalimab in crossover	27.7%	–	–	(14)
Others	47.2%	–	–	(14)
Discount rate	5%	0-8%	Fixed in PSA	–

SAEs, serious adverse events; PFS, progression-free survival; PD, progressive disease; PSA, probabilistic sensitivity analysis; sugemalimab group, sugemalimab + platinum-based chemotherapy; placebo group, placebo + platinum-based chemotherapy.

## Results

### Basic analysis

**Figure 2:**
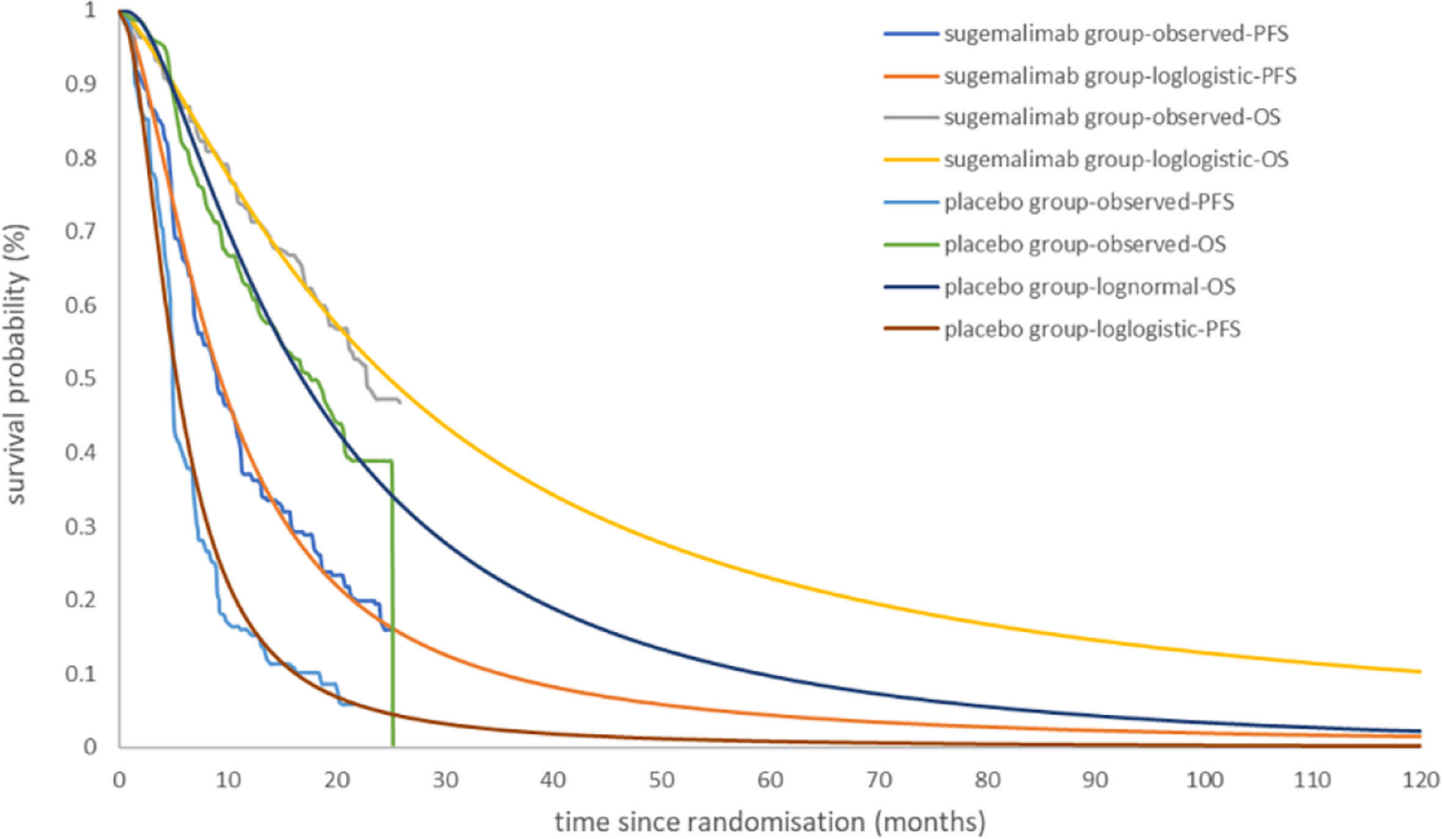
Kaplan-Meier Curve in the sugemalimab and placebo group using standard parametric models fitting and extrapolation.

The OS curve in the placebo group was best fit to a log-normal distribution. The PFS and OS curve in the sugemalimab group and the PFS curve in the placebo group were best fit to a log-logistic distribution (the curve fitting parameters were shown in **Table 2**, and the fitted curves were shown in **Figure 2**).

**Table 2:** Clinical Inputs: Kaplan-Meier survival curves Fitting Parameters.

	Best fitting	Parameters	Parameters	AIC	BIC
**Sugemalimab PFS**	Loglogistic	γ=1.6637	λ=9.3549	1567.99	1575.527
**Sugemalimab OS**	Loglogistic	γ=1.3722	λ=24.9818	1116.739	1124.275
**Placebo PFS**	Loglogistic	γ=1.9645	λ=5.3347	777.3102	783.448
**Placebo OS**	Lognormal	γ=2.8289	λ=0.9785	616.7406	622.8784

PFS, progression-free survival; OS, overall survival; AIC, Akaike information criterion; BIC, Bayesian information criterion; γ is the shape parameter and λ is the scale parameter.

The sugemalimab group could obtain 3.10 LYs, 1.63 QALYs, corresponding to a total cost of 130,667.70 USD; the placebo group can obtain 2.13 LYs, 1.04 QALYs, corresponding to a total cost of, 64,001.02 USD. the incremental cost per capita of the sugemalimab group compared with the placebo group was 66,666.63 USD, the incremental utility per capita was 0.59 QALYs and the ICER of 113,155.52 USD/QALY, which was much higher than the current WTP in China (36,203.88 USD/QALY), indicating that sugemalimab wasn’t economical for the first-line treatment of mNSCLC in combination with chemotherapy regimens at current prices (**Table 3**).

**Table 3:** Results.

	Cost (USD)	LYs	QALYs	Incremental cost (USD)	Incremental QALYs	ICER (USD/QALY)
Base-case analysis						
**Placebo group**	64001.02	2.13	1.04	–	–	–
**Sugemalimab group**	130667.70	3.10	1.63	66666.63	0.59	113155.52
Scenarios analysis 1						
The price of sugemalimab has been reduced by 70%						
**Placebo**	46425.22	2.13	1.04	–	–	–
**Sugemalimab**	80573.44	3.10	1.63	34148.22	0.59	57960.92
The price of sugemalimab has been reduced by 90%						
**Placebo**	41403.56	2.13	1.04	–	–	–
**Sugemalimab**	66260.78	3.10	1.63	24857.22	0.59	42190.99
Scenarios analysis 2						
5 years						
**Placebo group**	59126.99	1.96	0.97	–	–	–
**Sugemalimab group**	122481.10	2.5	1.39	63354.15	0.42	150265.63
20 years						
**Placebo group**	65461.93	2.19	1.06	–	–	–
**Sugemalimab group**	136483.20	3.49	1.78	71021.32	0.72	98650.85
Scenarios analysis 3						
Distribution of OS using Mixture cure model						
**Placebo group**	92219.17	3.16	1.37	–	–	–
**Sugemalimab group**	129034.80	3.00	1.60	36815.59	0.23	158853.40
Distribution of OS using Nonmixture cure model						
**Placebo group**	91173.42	3.13	1.35	–	–	–
**Sugemalimab group**	126415.10	2.85	1.55	35241.70	0.20	183110.90
Distribution of OS using Royston/Parmar spline model						
**Placebo group**	76526.42	2.6	1.19	–	–	–
**Sugemalimab group**	130667.10	3.1	1.63	54140.68	0.44	121742.30

LYs, life years; QALYs, quality-adjusted life years; ICER, incremental cost-effectiveness ratio; OS, overall survival; sugemalimab group, sugemalimab + platinum-based chemotherapy; placebo group, placebo + platinum-based chemotherapy.

### One-way sensitivity analysis

**Figure 3:**
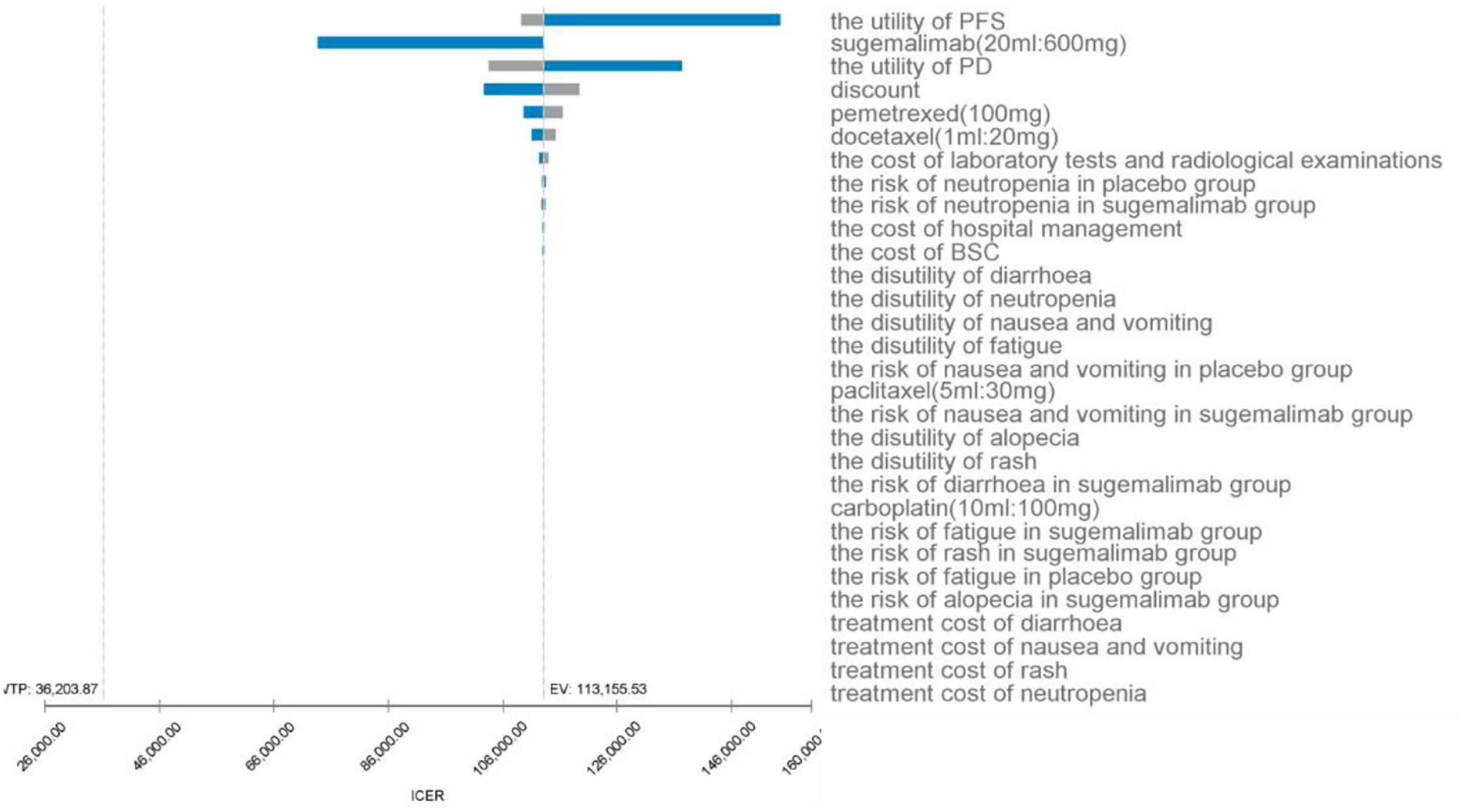
One-way sensitivity analysis for sugemalimab + platinum-based chemotherapy compared with placebo + platinum-based chemotherapy, PFS, progression-free-survival; PD, progressive disease; BSC, best supportive care; ICER, incremental cost-effectiveness ratio; SAE’s, serious adverse events; EV, expected value.

As can be seen from the tornado plot (**Figure 3**), the parameters that had the greatest impact on the results of the base analysis were the utility value of PFS, the price of sugemalimab, and the utility value of PD. The discount rate, the price of pemetrexed, the price of docetaxel, and the cost of laboratory and imaging tests during follow-up also had an impact on the model results. Parameters such as the cost of management of SAEs and cost during hospitalization had almost no effect on the results. Regardless of how the model parameters fluctuated within their upper and lower bounds, they did not affect the final results of the model, indicating that the model is somewhat robust.

### Probabilistic sensitivity analysis

**Figure 4:**
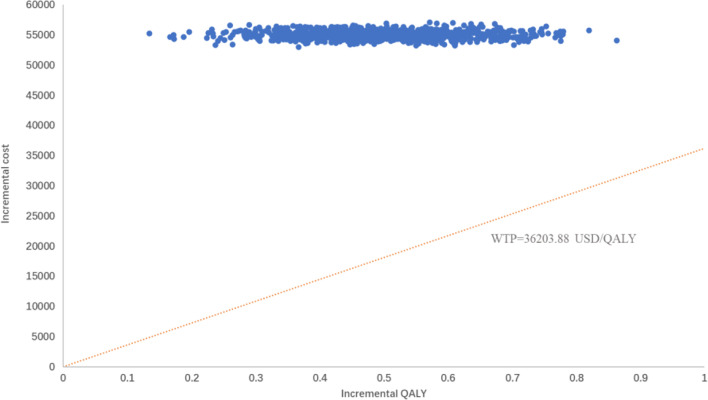
Probabilty sensitivity analysis scatter plot comparing sugemalimab + platinum-based chemotherapy and placebo + platinum-based chemotherapy. WTP willingness-to-pay, QALY quality-adjusted life-year.

**Figure 5:**
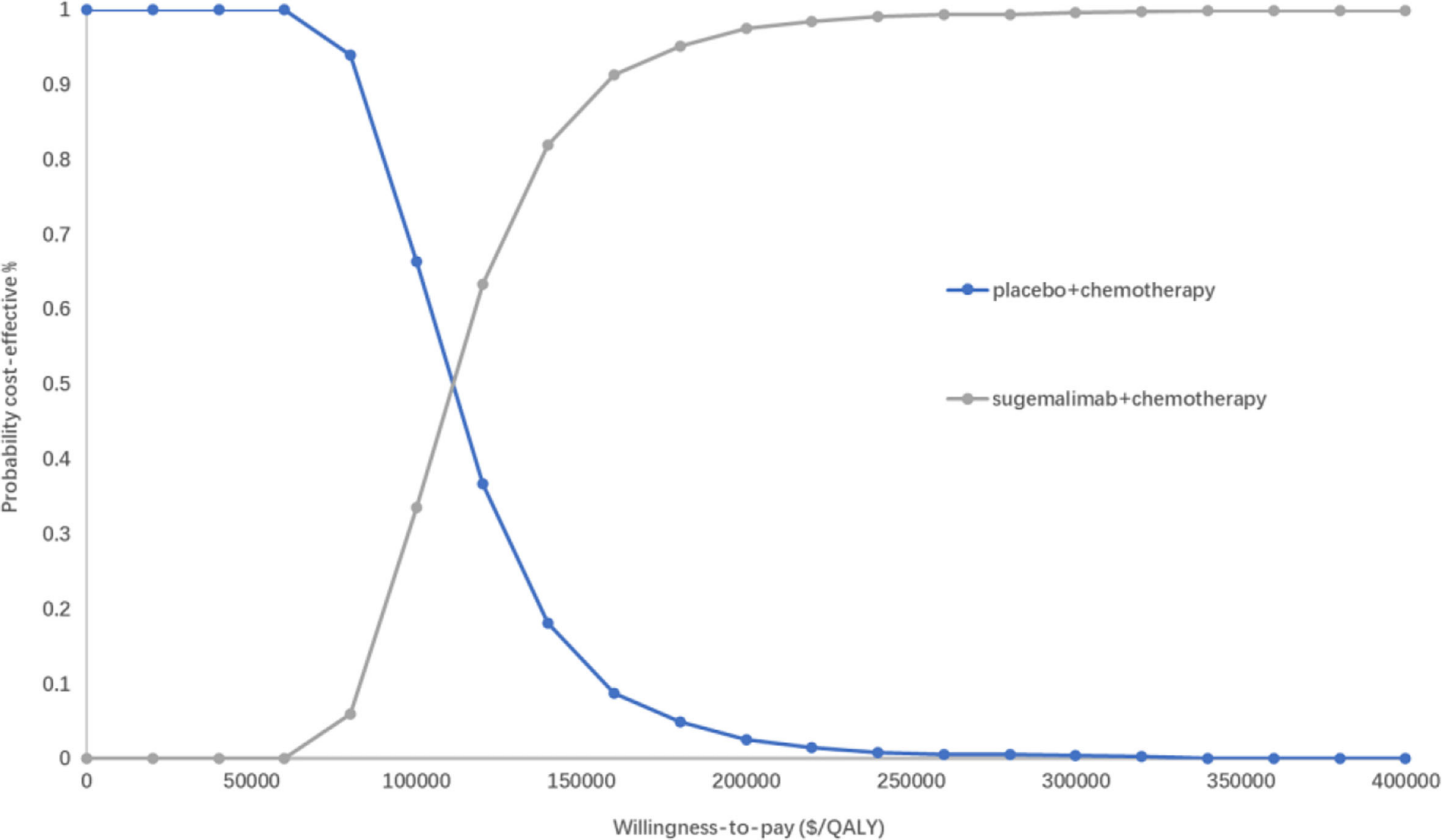
Cost-effectiveness acceptability curves of for sugemalimab + platinum-based chemotherapy versus placebo + platinum-based chemotherapy. QALY quality-adjusted life-year.

The PSA responded to the effect of overall changes in each parameter on the results. The incremental cost-effectiveness scatter plot (**Figure 4**) shows that almost all scatter points were distributed above the WTP; the CEAC shows (**Figure 5**) that the probability of sugemalimab plus chemotherapy having a cost-effective was 0 at the current WTP in China, and the probability of sugemalimab combination chemotherapy being economical was 100% when the WTP was set to 340,000 USD.

### Scenario analysis

Scenario analysis results showed (**Table 3**) that when the price of sugemalimab was reduced by 70% and 90%, the ICER of the sugemalimab group and placebo group were 57,960.92 USD/QALY and 42,190.99USD/QALY, respectively, which were still higher than the current WTP in China. This regimen was still not economical for the first-line treatment of mNSCLC in China.

When the running time of the model was set at 5 and 20 years, the incremental cost per capita of the sugemalimab group versus the placebo group was 63,354.15 USD and 71,021.32 USD, respectively; the incremental utility per capita was 0.42 QALYs and 0.72 QALYs, respectively; ICER was 150,265.63 USD/QALY and 98,650.85 USD/QALY, respectively.

**Figure 6:**
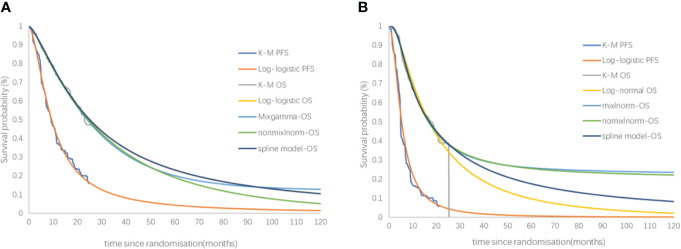
The fitting and extrapolation of the OS curves for the sugemalimab and the placebo group with the use of a mixture cure model, a nonmixture cure model, and the Royston/Parmar spline model. PFS, progression-free-survival, OS, overall survival. **(A)** PFS and OS of sugemalimab + platinum-based chemotherapy; **(B)** PFS and OS placebo + platinum-based chemotherapy.

The fitting and extrapolation of the OS curves for the sugemalimab and the placebo group with the use of a mixture cure model, a nonmixture cure model, and the Royston/Parmar spline model were shown in **Figure 6**. The OS curve was extrapolated using the mixture cure model, the sugemalimab group and the placebo group could obtain 1.60 and 1.37 QALYs, respectively, with an ICER of 158,853.40 USD/QALY. When the OS curve was extrapolated using the nonmixture cure model, the sugemalimab group and the placebo group could obtain 1.55 and 1.35 QALYs, respectively, with an ICER of 183,110.90.40 USD/QALY. when extrapolated using the Royston/Parmar spline model, 1.63 and 1.19 QALYs were obtained in the sugemalimab and placebo groups, respectively, with an ICER of 121,742.30 USD/QALY.

## Discussion

Since 1990, lung cancer incidence and mortality have been increasing due to smoking and environmental effects, resulting in the loss of about 24.9 million disability-adjusted life-years (DALYs), posing a serious threat to human health and a great challenge to global public health (22).

Traditional radiotherapy and chemotherapy have low safety and poor efficacy. In recent years, with the research on biomarkers of NSCLC, lung cancer has entered the era of “precision therapy”. The five-year survival rate of mNSCLC patients treated with new targeted and immune anti-tumor drugs has exceeded 20% (8, 9). Although targeted or immunotherapy has brought new hope to mNSCLC patients, the corresponding high economic burden is a new problem. How to ensure the availability of new antineoplastic drugs to patients while making the healthcare system sustainable is a very important issue. Therefore, reasonable pricing can improve the survival benefits of patients while reducing costs, and improve the availability of innovative drugs to patients and the enthusiasm of enterprises to develop new drugs. Especially at the present time when innovative drugs are changing with each passing day, how to choose the most reasonable drugs should take into account the patient’s indication and financial situation.

The economics of marketed PD-L1 inhibitors do not vary across countries and regions due to differences in drug prices and levels of socioeconomic development. The results of a study conducted from the perspective of the Chinese health system showed that the first-line treatment of squamous NSCLC with atezolizumab plus chemotherapy was not economical (23). In contrast, the results of a study conducted from the perspective of US health insurance payers showed that atezolizumab was cost-effective compared with pembrolizumab for first-line treatment of mNSCLC (24), and that the atezolizumab combination chemotherapy regimen was cost-effective for the first-line treatment of advanced NSCLC at a 43.3% price reduction for atezolizumab (25). A real-world retrospective study in Italy showed that atezolizumab was the most cost-effective compared to nivolumab and pembrolizumab for second-line treatment of advanced NSCLC (26), and that durvalumab was currently an economical option for consolidation therapy after radiotherapy for patients with stage III NSCLC, both from the perspective of the Chinese health system and from the perspective of US and Swiss health insurance payers (27–29).

This study evaluated the economics of sugemalimab plus chemotherapy in the first-line treatment of mNSCLC. The results of both the base-case and probabilistic sensitivity analysis indicated that the sugemalimab combination chemotherapy regimen was not economical in the current WTP. This result is generally consistent with the results of other PD-L1 studies that have been marketed in China: none of the PD-L1 inhibitors that have been marketed in China are economic when used in the first-line treatment of NSCLC. In addition to the high price of the immunotherapy drugs themselves, it may also be related to the low WTP setting. According to the Chinese pharmacoeconomic evaluation guidelines, we often taken 3 times the GDP per capita as the WTP, however, for major diseases like malignancy, the time spent by patients in the last stage of their lives is often more valuable to them as well as to society than the same amount of time in any other stage of their lives (30). In the United Kingdom and the United States, policies have been introduced for end-of-life treatment to raise the threshold of the society’s average WTP. In addition, the per capita economic level in China is unevenly developed, and the GDP varies greatly from region to region, and the per capita GDP of some regions is far below the national average, for example, the 3 times GDP per capita of Gansu Province and Yunnan Province is only $9,604.10 and $9,916.46, while the 3 times GDP per capita of Shenzhen and other economically developed regions in the Pearl River Delta is $84,421. Therefore, it is more reasonable to explore the payment standard for new antineoplastic drugs by combining the economic development level and the average WTP in China. At the same time, most of the current clinical trials or economic evaluations of PD-L1 inhibitors are comparisons of PD-L1 inhibitors combined with chemotherapy regimens compared to standard regimens such as chemotherapy regimens, and such comparisons are difficult to obtain an economic advantage. However, in the actual treatment, society, physicians and patients may be faced with the problem of choosing the “most appropriate” drug among many similar drugs with similar mechanisms. Therefore, there is an urgent need to conduct some “head-to-head” clinical trials to directly compare the efficacy and safety of new antitumor drugs and to provide a basis for subsequent economic evaluation.

Three different scenario analyses were set up in this study to better analyze the economics of sugemalimab in different application scenarios.

In scenario analysis 1, due to China’s health insurance negotiations and other related policies in recent years, many drug prices have seen significant decreases, so we compared here the economics of sugemalimab when it decreases by 70%-90%. Unfortunately, even if the price of sugemalimab is reduced by 90%, there is still a gap with the current WTP, which means that the clinical effectiveness and safety advantages of the sugemalimab combination chemotherapy regimen are still difficult to compensate for its lack of economics in the face of a significant decrease in overall treatment costs.

As shown in scenario 2, mNSCLC patients spent 90% of the total cost in the first five years after diagnosis, but the placebo group did not have a significant increase in incremental costs over 20 and 5 years compared with the sugemalimab group, and patients continued to benefit in subsequent survival over time, even after immune drug discontinuation, with a more significant advantage of incremental QALYs in the trial group. This may be related to a “delayed effect” due to the mechanism of action of the immunotherapy drugs. Therefore, the longer the study duration, the greater the clinical benefit and the better the economics of the regimen in the experimental group.

Scenario analysis 3 examinedthe effect of different curve fitting and extrapolation methods on the results. The standard parametric model was only suitable for survival extrapolation in uncomplicated situations. While conventional antitumor treatment drugs (e.g chemotherapy) exert their antitumor effects by directly attacking cancer cells, ICIs work by modulating the patient’s immune system and can induce a durable therapeutic response. Therefore, some patients still have therapeutic effects for several years after stopping treatment, and some patients are even cured and achieve long-term survival, which is expressed in the survival curve as a sustained “plateau period”. The more mature the data, the more accurate the extrapolation of survival curves. In practice, the limited follow-up time of clinical trials makes it difficult for short-term survival data to reflect the long-term survival benefits of ICIs. Over time, the patient population changes, the risks become more complex, and patients with immune responses become the only survivors whose basic patterns of survival have changed, making subsequent survival analysis more challenging. Some more flexible models are increasingly being used to extrapolate survival curves for ICIs, and studies have shown that Royston/Parmar spline model often underestimate long-term overall survival, and that mixture cure and nonmixture cure models extrapolate survival curves more closely to reality (31, 32).

In this study, three models other than the standard parametric model were used to extrapolate the OS curves of the sugemalimab group and the placebo group to investigate the impact of different survival curve extrapolation methods on economic assessment. The results showed that the Royston/Parmar spline model was close to the results of the standard parametric model. And the gap in survival benefit between the sugemalimab and chemotherapy groups narrowed when using the mixture cure model and the nonmixture cure model, resulting in a higher ICER, which may be related to the overrepresentation of patients in the chemotherapy group who continued treatment with sugemalimab after progression.

There are still limitations in this study: (1) The results of univariate sensitivity analysis also indicate that the utility value of PFS status has a large impact on the model results. There is a lack of health utility value studies specifically for Chinese NSCLC patients, and differences in the impact of the same disease on patients’ quality of life vary from countries to countries; (2) The cost of treatment after disease progression was calculated according to the current treatment guidelines for NSCLC using docetaxel, without taking into account individual differences, but in the actual clinical treatment setting, mNSCLC patients continued immunotherapy after progression of first-line treatment. 27.7% of patients in the placebo-combination chemotherapy group received sugemalimab after progression of first-line treatment, and these factors may narrow the gap in survival benefit between the two treatment regimens, resulting in a high final ICER.

Despite these limitations, the results of the basal analysis, one-way sensitivity analysis and PSA showed that the utility value and the cost of subsequent treatment would not affect the conclusion, demonstrating the robustness of the basal analysis. This study is still informative for health insurance policy access and rational clinical use of drugs.

## Conclusion

For systemically untreated mNSCLC, sugemalimab plus platinum-containing chemotherapy regimens have a higher survival benefit compared to standard chemotherapy regimens, but the medical costs are substantially higher and the regimen is not currently economical in China. An appropriate price reduction would increase the probability that it would be economical.

## Data availability statement

The original contributions presented in the study are included in the article/supplementary material. Further inquiries can be directed to the corresponding author.

## Author contributions

YZ was responsible for the design and conception of this study. HW and LL were mainly responsible for the construction of the model and the drafting of the article, while YX and YL participated in the collection of data and the drafting of the article. All authors contributed to the article and approved the submitted version.
